# Tripled likelihood: polypharmacy increases the occurrence of drug therapy problems in hospitalized pediatric patients

**DOI:** 10.3389/fphar.2024.1375728

**Published:** 2024-04-25

**Authors:** Bereket Takele, Hailu Chare Koyra, Temesgen Sidamo, Temesgen Leka Lerango

**Affiliations:** ^1^ School of Pharmacy, College of Health Sciences and Medicine, Wolaita Sodo University, Wolaita Sodo, Ethiopia; ^2^ School of Public Health, College of Health Sciences and Medicine, Dilla University, Dilla, Ethiopia

**Keywords:** drug therapy problem, DTP, polypharmacy, deprescribing, pediatrics, hospitalized, admitted, Ethiopia

## Abstract

**Background:**

A drug therapy problem (DTP) is any undesirable event experienced by a patient that accompanies drug therapy, prevents the patient from achieving their desired therapeutic goals, and requires expert judgment to resolve. Pediatric populations are at a higher risk of DTP than adults due to their immature organ systems, including the liver and kidneys, which play crucial roles in drug metabolism and excretion. Most previous studies have focused on only one element of DTP. Therefore, by considering all elements of DTP, we aimed to assess the prevalence of DTP and associated factors among pediatric patients admitted to the Wolaita Sodo University Comprehensive Specialized Hospital.

**Methods:**

An institution-based cross-sectional study was conducted among pediatric patients admitted to Wolaita Sodo University Comprehensive Specialized Hospital from 8 July 2020, to 7 July 2021. A simple random sampling technique was employed to select study participants. Cipolle’s and Strand’s classification method of drug therapy problems was used to identify and categorize DTP. Data were obtained by reviewing the patient’s medical records using a data abstraction checklist, entered into Epi data version 4.6, and exported to SPSS version 25 for analysis. Binary logistic regression analysis was performed to identify independent predictors of DTP.

**Results:**

Medical records of 369 pediatric patients were reviewed, and the overall prevalence of DTP was 60.2% (95% CI:55.2%, 65.2%) with a total of 281 identified DTPs. Among them, 164 (74.2%) had only one DTP. Need additional drug therapy was the most common (140 [49.8%]) DTP identified. The number of disease conditions (AOR = 2.13, 95% CI:1.16, 3.92), polypharmacy (AOR = 3.01, 95% CI:1.70, 5.32), and duration of hospital stay (AOR = 1.80, 95% CI:1.04, 3.10) were independent predictors of DTP among admitted pediatric patients.

**Conclusion:**

The prevalence of DTP in pediatric patients in the current setting was high. The number of disease conditions, polypharmacy, and duration of hospital stay were independent predictors of DTP. Enhancements to pharmaceutical care services, optimized dosage practices, improved deprescribing by clinicians, and efficient, comprehensive diagnostic procedures have the potential to significantly reduce specific drug therapy problems in hospitalized pediatrics.

## Introduction

A drug therapy problem (DTP) is any undesirable event experienced by a patient that accompanies drug therapy, prevents the patient from achieving their desired therapeutic goals, and requires expert judgment to resolve (Cipolle et al., 2012). Globally, DTP has increased significantly over the past few decades (Nivya et al., 2015). During the course of medication use, from prescription to follow-up treatment, DTP may occur at any time (Viktil and Blix, 2008; Cipolle et al., 2012). Worldwide, more than half of all medicines are prescribed and dispensed inappropriately, and half of the patients fail to take them correctly (World Health Organization, 2002; Bigdeli et al., 2015).

Unnecessary drug therapy, need additional drug therapy, ineffective drug therapy, dosage too low, adverse drug reactions, dosage too high, and non-adherence are seven types of drug therapy problems that fall under four patients’ drug-related needs (Cipolle et al., 2012). The risk of medication-related harm is three times greater for children than for adults (Kaushal et al., 2001). Each organ system, such as the liver and kidneys which play key roles in drug metabolism and excretion, is immature in the pediatric population (Maheshwari et al., 2019). The maturity of organ function and body composition can significantly impact the pharmacokinetics of various drugs (Kearns et al., 2003; Maheshwari et al., 2019). Medication errors are a significant problem for pediatric patients (Wong et al., 2009).

The higher likelihood of DTP among children is due to the different steps involved in calculating, reviewing, preparing, and administering doses, and the increased complexity in the approach to drugs used for children (Wong et al., 2009; Bereda, 2022). There is a greater risk of harm when pediatric medicines are optimized without robust trial data or appropriate medication formulations (Benn, 2014). To provide safe and effective drug therapy for children, it is imperative to understand and integrate the role growth and development plays in drug disposition and actions (Kearns et al., 2003). It is important to realize that absorption, distribution, metabolism, and excretion processes change throughout growth and development, so extrapolation can either lead to an overestimation or underestimation of the dose required for treatment (O'Hara et al., 2015).

Drug therapy problems have a significant impact on patient clinical outcomes, leading to reduced quality of life, higher morbidity and mortality, hospital admissions, long hospital stays, a considerable increase in the demand for additional drugs, and an increased healthcare cost burden on patients and the government (Easton et al., 2004; Viktil and Blix, 2008; Hoonhout et al., 2009; Elliott et al., 2018). Different studies have revealed a considerable proportion of drug-related visits and hospitalizations, although the majority are preventable (Commission, 2009; Braun et al., 2012; Al-Azzam et al., 2016; Feyissa et al., 2020).

Various factors contribute to the occurrence of DTP. These include polypharmacy, certain infectious and parasitic diseases, length of hospital stay, number of diseases, and number of drugs per patient (Rashed et al., 2014; Birarra et al., 2017; Meknonnen et al., 2017). It is critical to detect and classify DTP to provide relevant solutions and achieve desired outcomes at the lowest possible cost (Gelchu and Abdela, 2019). The remedies for identified DTPs include changing drug products or their doses, educating the patients on how to maximize medication effectiveness, and developing a care plan of individualized goals of therapy for each patient (Hepler and Strand, 1990).

Most previous studies have focused on one element of DTP, such as adverse drug reactions (ADRs) or dosing. Consequently, the findings of such investigations are insufficient to portray the full picture of DTP. Moreover, evidence of DTP among admitted pediatric patients is limited in low-income settings. Therefore, the current study aimed to assess the prevalence of DTP and associated factors among pediatric patients admitted to WSUCSH by considering all aspects of DTP. The findings of this study will benefit patients, practitioners, health institutions, and policymakers. It can also be used as a tool to empower pharmaceutical care services and promote the importance of clinical pharmacists in the hospital’s pediatric units.

## Materials and methods

### Study setting

This study was conducted in the pediatric ward of the Wolaita Sodo University Comprehensive Specialized Hospital (WSUCSH). The hospital has 437 beds for inpatient care. It is located in the Wolaita zone 329 km from Addis Ababa, the capital of Ethiopia. The hospital was established in 1928 and is one of the comprehensive specialized hospitals in the region providing services to 3.5–5 million people annually for patients from Wolaita, Dawuro, Gamo, Gofa, and Kambata Tambaro. Among the different departments in the hospital, the pediatric ward has 38 beds and provides inpatient services to 900 pediatric patients annually. Six senior pediatricians and other dedicated staff from various disciplines serve in the pediatric ward.

### Study design and period

A retrospective cross-sectional study was conducted at WSUCSH from 8 July 2020, to 7 July 2021, reviewing patient charts and physicians’ medication orders for children admitted to the pediatric ward.

### Population of the study

Pediatric patients admitted to the pediatric wards at WSUCSH who stayed for more than 48 h and received any type of drug were included, while patients with incomplete background information, those receiving maintenance fluids only, and those receiving blood transfusions only were excluded.

### Sample size determination

The sample size was calculated using a single-population proportion formula based on the following assumptions: *p* = 31.57%, prevalence of drug therapy problems among pediatric patients from a previous study conducted at Zewditu Memorial Referral Hospital, Addis Ababa, Ethiopia (Birarra et al., 2017), 95% confidence level, 5% degree of precision, and z-value at 95% confidence level of 1.96. The sample size calculated based on the above considerations was 332, and with anticipation of 10% incomplete or poor-quality records, the final sample size determined for this study was 369.

### Sampling procedure

The sampling frame was constructed using the patient’s discharge registration book for the study period of 1 year from 08 July 2020, to 07 July 2021. The card numbers of all admitted pediatric patients over 1 year are listed and used as a sampling frame. A simple random sampling technique was employed to select the card number of the study participants. All relevant information was extracted from the medical records of randomly selected patients.

### Operational definitions

#### Drug therapy problem

Any undesirable event experienced by a patient that involves, or is suspected to involve drug therapy and that interferes with achieving the desired goals of therapy and requires professional judgment to resolve (Pharmaceutical Care Network Europe Foundation, 2010).

#### Polypharmacy

Concomitant use of five or more prescription medications (World Health Organization, 2019).

#### Unnecessary drug therapy

Drug therapy is unnecessary because the patient does not have a clinical indication (Cipolle et al., 2012).

#### Need for additional drug therapy

Additional drug therapy is required to treat or prevent a medical condition or illness from developing, or the clinical condition requires initiation of drug therapy (Pharmaceutical Care Network Europe Foundation, 2010).

#### Ineffective drug

The drug product is ineffective at producing the desired response, or the medical condition is refractory to the drug product (Pharmaceutical Care Network Europe Foundation, 2010).

#### Inappropriate dosage

Refers to dosages that are both too low and too high (Pharmaceutical Care Network Europe Foundation, 2010).

• Dosage too high: indicates that the dose is too high and results in undesirable effects.
• Dosage too low: refers to the dose being too low to produce the desired response.

### Procedure for DTP identification and classification

The DTP evaluation tool was prepared based on Cipolle’s and Strand’s DTPs category classification system (Cipolle et al., 2012). This classification system is a widely accepted patient-centered textbook, which is the standardized guideline for pharmacists while practicing pharmaceutical care services and authorized by the Ethiopian Hospital Reform Implementation Guidelines (EHRIG) and Pharmaceuticals Fund and Supply Agency (PFSA) to implement the provision of pharmaceutical care services in Ethiopian hospitals (FDRE Ministry of Health, 2010; Pharmaceuticals Fund and Supply Agency, 2015).

Drug therapy problems were identified by reviewing and analyzing all medication orders, administration sheets, and laboratory and diagnostic test results, then DTPs were identified by evaluating the appropriateness of prescriptions in terms of indication, dosage, duration of therapy, appropriateness of drug choice using the Pocketbook of Pediatric Hospital Care (World Health Organization, 2013), Ethiopian Standard Treatment Guidelines 2021, Nelson textbook of Pediatrics 21st edition, and different therapeutic guidelines. Medscape drug interaction checkers and other convenient instruments were used to assess drug interactions.

The identified DTPs were classified as unnecessary drug therapy, need for additional drug therapy, ineffective drug therapy, dosage too low, dosage too high, adherence, and adverse drug reactions.

### Data collection method

Relevant information about each patient, including demographic data, patients’ clinical characteristics, physical examination, laboratory results, current medications, comorbidities, and relevant previous medical and medication histories were obtained from the medical records. The data extraction format included patient details, investigations, current and past medications, daily doses, comorbidities and their management, duration, and treatment targets. Two junior pharmacists and one senior pharmacist were assigned for data collection and supervision, respectively.

### Data quality assurance

The principal investigator trained the data collectors and supervisors on the study’s aim, strict adherence to the data abstraction format, and how and what data were collected from the medical records. To ensure the quality of the data, the data abstraction format was pretested before the main data collection on 18 randomly selected patient medical charts (5% of the total sample size) in Sodo Christian Hospital, and appropriate adjustments were made to the data collection format. To ensure completeness and consistency, the supervisor checked the collected data daily.

### Data processing and analysis

The data were entered using Epi-data version 4.6 and analysed using SPSS version 25. Descriptive statistics were used to describe the results of the study participants and characterize the DTPs. Frequencies and percentages were used to describe categorical variables, while mean and standard deviations for continuous data. The strength of the association between DTP and the explanatory variables was estimated using a binary logistic regression model. For multivariable logistic regression analysis, candidate variables were identified using bivariable logistic regression analyses. Variables with a *p*-value <0.25 in the bivariable regression analyses were candidates for multivariable regression analysis. Finally, those variables with a *p*-value <0.05 in multivariable analysis were declared as significant predictors of drug therapy problems.

## Results

**FIGURE 1:**
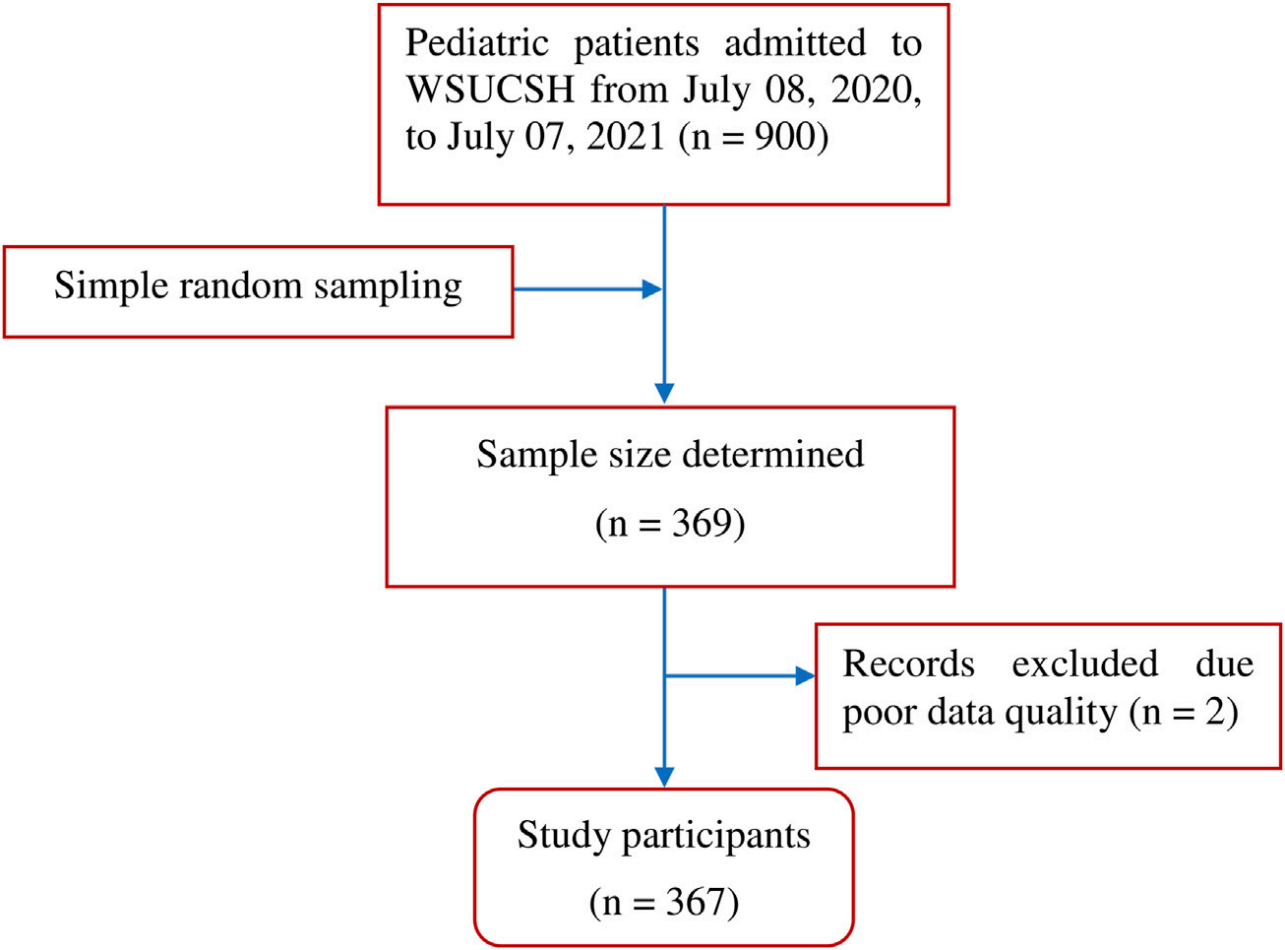
Flow diagram of sample selection and participation.

The medical records of 369 pediatric patients admitted to the WSUCSH between 8 July 2020, and 7 July 2021, were reviewed, and two records were excluded because of poor data quality (Figure 1).

### Baseline characteristics

#### Socio-demographic characteristics

The mean (±SD) age of the study participants was 3.83 (±4.39) years. Two-thirds of the study participants (138 [37.6%]) were infants, and more than half (202 [55%]) were males. The mean (±SD) weight was 13.28 (±9.74) kg. More than a third of participants (142 [38.7%]) weighed between five and 9.99 kg (Table 1).

**TABLE 1:** Socio-demographic characteristics of pediatric patients admitted to WSUCSH from 8 July 2020, to 7 July 2021 (n = 367).

Variables	Frequency (n)	Percent (%)
Sex		
Male	202	55
Female	165	45
Age		
Neonate (birth to 28 days)	7	1.9
Infant (29 days to ≤1 year)	138	37.6
Toddler (>1 to ≤3years)	61	16.6
Preschool (>3 to ≤5 years)	43	11.7
School-age (>5 to ≤10 years)	58	15.8
Adolescent (>10 to ≤16years)	60	16.3
Weight (in kg)		
<5	29	7.9
5–9.99	142	38.7
10–14.99	90	24.5
15–19.99	33	9.0
20–24.99	23	6.3
≥25	50	13.6

#### Clinical characteristics

**FIGURE 2:**
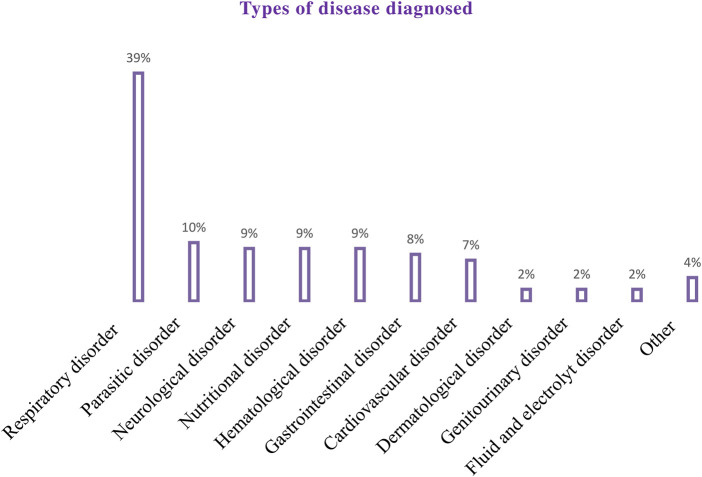
Types of disease diagnosed among pediatric patients admitted to the WSUCSH from 8 July 2020, to 7 July 2021.

The majority (302 [82.3%]) of the patients included in the study had comorbidities. More than a third of the participants (144 [39.2%]) stayed six to 10 days in the hospital. Approximately three-fourths of the patients (266 [72.5%]) were admitted to the inpatient unit (Table 2). A total of 864 diseases were diagnosed in all study participants. Of those, the most common disease diagnosed during the study period was the respiratory system (340 [39%]) (Figure 2).

**TABLE 2:** Clinical characteristics of pediatric patients admitted to WSUCSH from 8 July 2020, to 7 July 2021 (n = 367).

Variables	Frequency (n)	Percent (%)
Comorbidity		
Yes	302	82.3
No	65	17.7
Duration of hospital stay		
<6 days	133	36.2
6–10 days	144	39.2
>10 days	90	24.5
Number of diagnoses		
1	65	17.7
2	165	45
3	87	23.7
≥4	50	13.6
Admission ward		
SAM	57	15.5
Inpatient	266	72.5
ICU	44	12
Complete blood count done		
Yes	343	93.5
No	24	6.5

#### Medication-related characteristics

**FIGURE 3:**
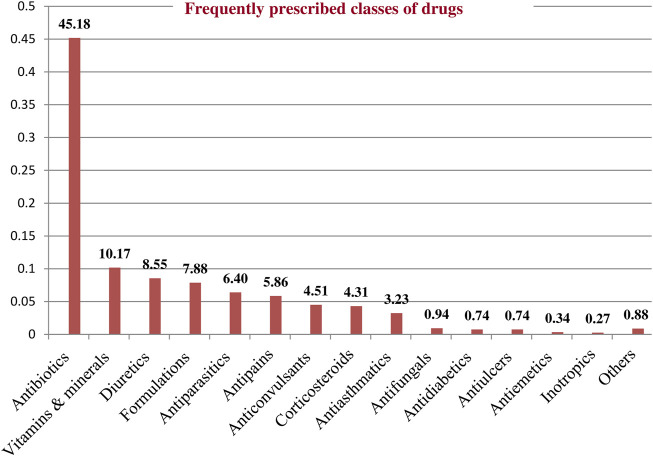
Frequently prescribed classes of drugs among pediatric patients admitted to WSUCSH from 8 July 2020, to 7 July 2021.

A total of 1,485 drugs were prescribed to 367 patients during the study period. The mean (±SD) number of drugs per patient was 4.22 (±1.8). The most commonly prescribed classes of drugs were Antibiotics followed by Vitamins and Minerals, and Diuretics (Figure 3). Among the study participants, the majority (226 [61.6%]) received five or more medicines.

### Types and prevalence of drug therapy problems

**FIGURE 4:**
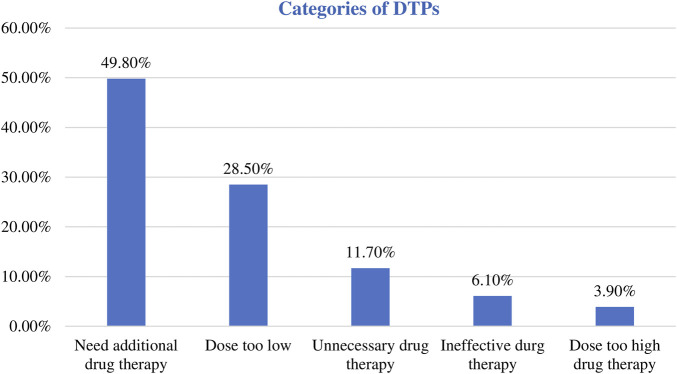
Categories of the identified DTPs among pediatric patients admitted to WSUCSH from 8 July 2020, to 7 July 2021.

Among the 367 participants, 221 had encountered at least one DTP, resulting in a prevalence of 60.2% (95% CI:55.2%, 65.2%). A total of 281 DTPs were identified. The mean (±SD) number of DTP per patient was 1.27 (±0.48). The maximum number of DTPs per patient was three. Most patients (164 [74.2%]) had only one DTP (Table 3). Among the DTPs identified, ‘need additional drug therapy’ was found to be the most frequent DTP (140 [49.8%]) followed by ‘dose too low’ (80 [28.5%]) (Table 4) (Figure 4).

**TABLE 3:** Total number of drug therapy problems per patient among pediatric patients admitted to WSUCSH from 8 July 2020, to 7 July 2021.

No.	No. of patients	No. of DTPs	Total no of DTPs	% of patients with DTP
1	164	Only one DTP per patient	164	74.2
2	54	Two DTPs per patient	108	24.4
3	3	Three DTPs per patient	9	1.4
Total	221		281	100

**TABLE 4:** The common reasons for DTPs identified among pediatric patients admitted to WSUCSH from 8 July 2020, to 7 July 2021 (n = 281).

DTP category and cause	Frequency (%)
Need additional drug therapy	140 (49.8)
A medical condition requires the initiation of a drug	123 (87.86)
Preventive drug therapy is required	17 (12.14)
Dose too low	80 (28.5)
Dose is too low to produce the desired response	25 (31.25)
Duration of drug therapy is too short	6 (7.5)
Dosage interval is too infrequent	49 (61.25)
Unnecessary drug therapy	33 (11.7)
No valid medical indication	14 (42.42)
Multiple drug products are being used	19 (57.58)
Ineffective drug therapy	17 (6.1)
The least effective drug is used	16 (94.12)
Drug product is not an effective product	1 (5.88)
Dose too high	11 (3.9)
Dose is too high	7 (63.64)
Dosing frequency is too short	1 (9.09)
Duration of drug therapy is long	3 (27.27)

### Factors associated with drug therapy problems

In the bivariable logistic regression analyses, age, duration of hospital stay, comorbidity, polypharmacy, number of disease conditions, weight, and admission ward were found to be candidates for multivariable logistic regression analysis (Table 5).

**TABLE 5:** Bivariable logistic regression analysis of factors associated with drug therapy problems among pediatric patients admitted to WSUCSH from 8 July 2020, to 7 July 2021 (n = 367).

Variables	Category	DTP		COR (95% CI)	*p*-value
		Yes	No		
Sex					
	Male	121(59.9)	81(40.1)	0.97 (0.64, 1.48)	0.891
	Female	100(60.6)	65(39.4)	1	
Age					
	Neonate	6(85.7)	1(14.3)	4.59 (0.52, 40.50)	0.170*
	Infant	90(65.2)	48(34.8)	1.43 (0.77, 2.66)	0.254
	Toddler	30(49.2)	31(50.8)	0.74 (0.36, 1.51)	0.410
	Preschool	25(58.1)	18(41.9)	1.06 (0.48, 2.35)	0.882
	School-age	36(62.1)	22(37.9)	1.25 (0.60, 2.61)	0.551
	Adolescent	34(56.7)	26(43.3)	1	
Duration of hospital stay (in days)					
	<6	62(46.6)	71(53.4)	1	
	6–10	94(65.3)	50(34.7)	2.15 (1.33, 3.49)	0.002*
	>10	65(72.2)	25(27.8)	2.98 (1.68, 5.28)	0.000*
Comorbidity					
	Yes	186(61.6)	116(38.4)	1.37 (0.80, 2.36)	0.248*
	No	35(53.8)	30(46.2)	1	
Polypharmacy					
	Yes	123(79.9)	31(20.1)	4.66 (2.89, 7.50)	0.000*
	No	98(46.0)	115(54.0)	1	
Number of disease conditions					
	<3	114(49.6)	116(50.4)	1	
	≥3	107(78.1)	30(21.9)	3.63 (2.25, 5.87)	0.000*
CBC done					
	Yes	205(59.8)	138(40.2)	0.74 (0.31, 1.78)	0.506
	No	16(66.7)	8(33.3)	1	
Admission ward					
	SAM	45(78.9)	12(21.1)	1.10 (0.43, 2.85)	0.840
	Inpatient	142(53.4)	124(46.6)	0.34 (0.16, 0.71)	0.004*
	ICU	34(77.3)	10(22.7)	1	

Bold value represent *p*-value <0.25.

In multivariable logistic regression, the number of disease conditions, polypharmacy, and duration of hospital stay were independent predictors of drug therapy problems among admitted pediatric patients. Accordingly, those patients who had three or more disease conditions were twice as likely to encounter DTP in comparison with patients who had less than three disease conditions (AOR = 2.13, 95% CI:1.16, 3.92). Polypharmacy was also found to be a statistically significant predictor of DTP. The likelihood of DTP was three times higher for patients with polypharmacy than for those without (AOR = 3.01, 95% CI:1.70, 5.32). Similarly, the odds of encountering DTP were about two-fold higher for patients who stayed for 6–10 days on admission than for those who stayed for less than 6 days (AOR = 1.80, 95% CI:1.04, 3.10) (Table 6).

**TABLE 6:** Multivariable logistic regression analysis of factors associated with drug therapy problems among pediatric patients admitted to WSUCSH from 8 July 2020, to 7 July 2021 (n = 367).

Variables	Category	DTP		AOR (95% CI)	*p*-value
		Yes	No		
Age					
	Neonate	6(85.7)	1(14.3)	8.98 (0.92, 87.56)	0.059
	Infant	90(65.2)	48(34.8)	1.76 (0.88, 3.53)	0.113
	Toddler	30(49.2)	31(50.8)	0.97 (0.42, 2.24)	0.937
	Preschool	25(58.1)	18(41.9)	1.17 (0.48, 2.84)	0.738
	School-age	36(62.1)	22(37.9)	1.53 (0.66, 3.54)	0.324
	Adolescent	34(56.7)	26(43.3)	1	
Duration of hospital stay (in days)					
	<6	62(46.6)	71(53.4)	1	
	6–10	94(65.3)	50(34.7)	1.80 (1.04, 3.10)	0.036*
	>10	65(72.2)	25(27.8)	1.54 (0.76, 3.12)	0.232
Comorbidity					
	Yes	186(61.6)	116(38.4)	0.76 (0.41, 1.43)	0.398
	No	35(53.8)	30(46.2)	1	
Polypharmacy					
	Yes	123(79.9)	31(20.1)	3.01 (1.70, 5.32)	0.000*
	No	98(46.0)	115(54.0)	1	
Number of disease conditions					
	<3	114(49.6)	116(50.4)	1	
	≥3	107(78.1)	30(21.9)	2.13 (1.16, 3.92)	0.015*
Admission ward					
	SAM	45(78.9)	12(21.1)	1.50 (0.52, 4.37)	0.457
	Inpatient	142(53.4)	124(46.6)	0.67 (0.29, 1.58)	0.365
	ICU	34(77.3)	10(22.7)	1	

Bold value represent statistically significant association at *p*-value <0.05.

## Discussions

Prior studies have assessed drug therapy problems, focusing mainly on one aspect. However, the current study at the WSUCSH estimated the prevalence of drug therapy problems in admitted pediatric patients by assessing most of its elements. Investigation of DTP considering most of its elements will assist in developing a holistic intervention approach to counter the occurrence of DTP among admitted pediatric patients. In this study, 281 DTPs were identified resulting in the overall prevalence of DTP to be 60.2%. Moreover, the number of disease conditions, polypharmacy, and duration of hospital stay were independent predictors of drug therapy problems.

The overall prevalence of drug therapy problems was 60.2% (95% CI:55.2%, 65.2%). This finding illustrates that a substantial number of pediatric patients admitted to the WSUCSH have encountered drug therapy problems. The prevalence of DTP in the current setting is higher than that in previous studies conducted at Zewdtu Memorial Referral Hospital in Addis Ababa, KAMC-Jeddah, and Hong Kong, which reported DTP prevalence rates of 31.57%, 35.9%, and 21%, respectively (Rashed et al., 2014; Birarra et al., 2017; AlAzmi et al., 2019). DTPs were classified using different methods, which may account for this difference. We employed Cipolle’s classification method, while studies in Addis Ababa (Birarra et al., 2017) and Hong Kong (Rashed et al., 2014) employed the Pharmaceutical Care Network Europe (PCNE) classification system. Moreover, the observed difference might be due to disparities in the healthcare systems and practitioners across countries.

Regarding the categories of DTPs, need additional drug therapy was the most common DTP in this investigation, accounting for 50% of the total DTPs. This finding regarding the need for additional drug therapy for DTP is higher than that reported in previous studies in Ethiopia. A study in Mettu Karl Referral Hospital (MKRH) reported 25.09% (Bekele et al., 2021), whereas another study in a pediatric ward of Dessie Referral Hospital reported 25.2% (Bizuneh et al., 2020). This is also higher than that reported in Hong Kong (3.7%) (Rashed et al., 2014). A possible explanation for this difference could be the variation in the level of knowledge and experience of the health professionals at these health facilities concerning the appropriate indication for drugs and the proper choice of drugs based on observed cases. Furthermore, comorbidities and patient conditions may have contributed to these differences.

In the current investigation, dose too low was responsible for 28.5% of the identified DTPs. This was higher than the results reported in Brazilian and Hong Kong studies, which found doses too low to be 13.1% and 19.5%, respectively (Rashed et al., 2014; Nascimento et al., 2020). This finding is consistent with that of a prospective observational study conducted at Jimma University Medical Center (JUMC), which reported 27.52% (Feyissa Mechessa et al., 2020). This study showed that low doses were more common in hospitalized pediatric patients, which might be due to improper weight-based dose calculations and frequency of administration. On the other hand, the DTP category identified in this study, the 3.9% dose too high, which is lower than the 15.9% and 16.1% findings from studies conducted in Hong Kong and Saudi Arabia, respectively (Rashed et al., 2014; AlAzmi et al., 2019).

In this study, unnecessary drug therapy was 11.7%, which aligns with the 16.8% reported in a study conducted in the pediatric ward of Dessie Referral Hospital, Ethiopia (Bizuneh et al., 2020). This showed that a considerable proportion of patients were given unnecessary medications, which might have resulted in adverse drug reactions and additional medication-related costs. Therefore, it is worthwhile to avoid unnecessary drug therapy and improve deprescribing practices to improve treatment outcomes in hospitalized patients. In the present study, 6.1% of the DTPs were found to be ineffective drug therapy. This is in line with that of Dessie’s study, 2.5% (Bizuneh et al., 2020), but lower than the 49% reported in the Brazilian study among cardiac neonates under intensive care (Nascimento et al., 2020).

As revealed in this study, the duration of hospital stay was significantly associated with drug therapy problems, with higher odds of drug therapy problems among pediatric patients who stayed 6–10 days hospitalized. This is consistent with Ethiopian studies conducted at Jimma University Medical Center, Metu Karl Referral Hospital, and Nekemte Referral Hospital (Dedefo et al., 2016; Feyissa Mechessa et al., 2020; Bekele et al., 2021). A possible explanation is that the more patients stay in the hospital, the greater the risk of hospital-acquired (nosocomial) infections, making drug regimens more complex. Complex drug regimens are risk factors for administration errors and nonadherence (Pantuzza et al., 2017; Schmidt et al., 2020); thus, early and accurate identification of the patient’s condition is crucial for reducing hospital stay. Efficient and comprehensive diagnostic procedures enable healthcare professionals to achieve this goal. In addition, adherence to the recommended treatment guidelines while prescribing the drug frequency and dose would be worthwhile.

A significant association was also found between polypharmacy and DTP. The likelihood of DTP was three times higher in patients with polypharmacy than in their counterparts. This finding is in agreement with the study conducted in Addis Ababa and Jimma on DTP, which reported that patients on polypharmacy have a complex drug schedule that may contribute to poor medication compliance, adverse drug effects, potential drug-drug interactions, and increased risk of DTP occurrence (Birarra et al., 2017; Feyissa Mechessa et al., 2020). Primary care physicians can address problems of polypharmacy by considering deprescribing, a set of interventions to identify inappropriate or unnecessary medications, and discontinuing them, as an essential part of good prescribing (Endsley, 2018; Farrell and Mangin, 2019). The involvement of clinical pharmacists in multidisciplinary care teams may lead to better treatment outcomes by providing informed decision-making.

In the present study, the number of disease conditions was an independent predictor of DTP. Those who had three or more disease conditions were two times more likely to encounter DTP than those who had fewer conditions. This is corroborated by previous studies on DTP that have identified the number of disease conditions as major predictors of DTP (Feyissa Mechessa et al., 2020; Bekele et al., 2021). The more health conditions you have, the more likely you are to take more than one type of prescription drug to manage them. There is a risk of drug-drug or drug-disease interactions. These interactions could lower the effectiveness of the drug in patients.

Improved pharmaceutical care services, which engage clinical pharmacists in the study setting, might minimize drug therapy problems in the pediatric population. An appropriate type of medication and prescribed doses may be tailored to the patient’s needs when clinical pharmacists collaborate with prescribing physicians. Optimizing medication dosages for the pediatric population could significantly reduce certain identified DTPs and this could be a prospect of our study.

## Strengths and limitations of the study

The current study used a standardized DTP identification tool that was evaluated and accepted by experts, but it is not without limitations. Although we attempted to investigate all categories of drug therapy problems, adverse drug reactions, and patient adherence were not included because of the retrospective study design constraints and poor documentation trends in the current setting for these two categories of DTPs. The retrospective design employed in the current study did not allow for the assessment of two categories of DTPs. This limitation might have affected the overall observed prevalence of DTPs.

Furthermore, since the study was conducted at a single center, WSUCSH, it may not have successfully represented the diversity of the population that could have been achieved in a multicenter setting. This limitation potentially affects the generalizability of the study’s results. Whenever practitioners translate the current findings to their setting, they should carefully consider limitations to the external validity that may arise from a single-center study. Therefore, future studies should consider a multicenter prospective study design that enables the inclusion of a diverse population and comprehensive data collection across all categories of DTPs.

## Conclusion

The current study showed that the majority of pediatric patients admitted to the WSUCSH had at least one drug therapy problem, indicating that optimal medication management in pediatric patients remains a major challenge in clinical practice. Need additional drug therapy was the most common DTP identified followed by a dose too low. The number of disease conditions, polypharmacy, and duration of hospital stay were independent predictors of drug therapy problems. Enhancements to pharmaceutical care services, optimized dosage practices, improved deprescribing by clinicians, and efficient, comprehensive diagnostic procedures have the potential to reduce specific drug therapy problems in hospitalized pediatrics significantly.

## Data Availability

The raw data supporting the conclusion of this article will be made available by the authors, without undue reservation.

## Abbreviations

ADR, Adverse Drug Reaction; DTP, Drug therapy Problem; ED, Emergency Department; HIV, Human Immunodeficiency Virus; NICU, Neonatal Intensive Care Unit; PCNE, Pharmaceutical Care Network Europe; PICU, Pediatric Intensive Care Unit; SAM, Severe Acute Malnutrition; WSUCSH, Wolaita Sodo University Comprehensive Specialized Hospital.

## References

[B1] Alazmi A. Ahmed O. Alhamdan H. Algarni H. Elzain R. M. Althubaiti R. S. (2019). Epidemiology of preventable drug-related problems (DRPs) among hospitalized children at KAMC-Jeddah: a single-institution observation study. Drug, Healthc. patient Saf. 11, 95–103. 10.2147/DHPS.S220081 31819660 PMC6886556

[B2] Al-Azzam S. I. Alzoubi K. H. Aburuz S. Alefan Q. (2016). Drug-related problems in a sample of outpatients with chronic diseases: a cross-sectional study from Jordan. Ther. Clin. risk Manag. 12, 233–239. 10.2147/TCRM.S98165 26937195 PMC4762438

[B3] Bekele F. Bereda G. Tamirat L. Geleta B. A. Jabessa D. (2021). Childrens are not just “little adults”. The rate of medication related problems and its predictors among patients admitted to pediatric ward of southwestern Ethiopian hospital: a prospective observational study. Ann. Med. Surg. 70, 102827. 10.1016/j.amsu.2021.102827 PMC8435910 34540216

[B4] Benn C. E. (2014). Optimising medicines for children: considerations for clinical pharmacists. Eur. J. Hosp. Pharm. 21, 350–354. 10.1136/ejhpharm-2013-000396

[B5] Bereda G. (2022). Pediatrics pharmacokinetics and dose calculation. J. Pediatr. Neonatal. Cares 12, 96–102. 10.15406/jpnc.2022.12.00463

[B6] Bigdeli M. Laing R. Tomson G. Babar Z.-U.-D. (2015). Medicines and universal health coverage: challenges and opportunities. J. Pharm. policy Pract. 8, 8–3. 10.1186/s40545-015-0028-4 25825675 PMC4350289

[B7] Birarra M. K. Heye T. B. Shibeshi W. (2017). Assessment of drug-related problems in pediatric ward of Zewditu memorial referral hospital, Addis Ababa, Ethiopia. Int. J. Clin. Pharm. 39, 1039–1046. 10.1007/s11096-017-0504-9 28689305 PMC6206436

[B8] Bizuneh G. K. Adamu B. A. Bizuayehu G. T. Adane S. D. (2020). A prospective observational study of drug therapy problems in pediatric ward of a referral hospital, Northeastern Ethiopia. Int. J. Pediatr. 2020, 4323189. 10.1155/2020/4323189 32256614 PMC7115047

[B9] Braun L. Sood V. Hogue S. Lieberman B. Copley-Merriman C. (2012). High burden and unmet patient needs in chronic kidney disease. Int. J. Nephrol. renovascular Dis. 5, 151–163. 10.2147/IJNRD.S37766 PMC3534533 23293534

[B10] Cipolle R. J. Strand L. M. Morley P. C. (2012). Pharmaceutical care practice: the patient-centered approach to medication management services. United States: McGraw-Hill Medical.

[B11] Commission H. (2009). Investigation into mid staffordshire NHS foundation trust. London: Healthcare Commission.

[B12] Dedefo M. G. Mitike A. H. Angamo M. T. (2016). Incidence and determinants of medication errors and adverse drug events among hospitalized children in West Ethiopia. BMC Pediatr. 16, 81–10. 10.1186/s12887-016-0619-5 27387547 PMC4936294

[B13] Easton K. L. Chapman C. B. Brien J. a. E. (2004). Frequency and characteristics of hospital admissions associated with drug-related problems in paediatrics. Br. J. Clin. Pharmacol. 57, 611–615. 10.1111/j.1365-2125.2003.02052.x 15089814 PMC1884507

[B14] Elliott R. Camacho E. Campbell F. Jankovic D. St James M. M. Kaltenthaler E. (2018). Prevalence and economic burden of medication errors in the NHS in England: rapid evidence synthesis and economic analysis of the prevalence and burden of medication error in the UK. Policy Research Unit in Economic Evaluation of Health and Care Interventions.

[B15] Endsley S. (2018). Deprescribing unnecessary medications: a four-Part Process. Fam. Pract. Manag. 25, 28–32. 29989773

[B16] Farrell B. Mangin D. (2019). Deprescribing is an essential part of good prescribing. Am. Fam. Physician 99, 7–9. 30600973

[B17] Fdre Ministry of Health (2010). Ethiopian hospital Reform implementation guidelines: vol-2. Addis Ababa, Ethiopia: Ethiopian Hospital Management Initiative May.

[B18] Feyissa D. Kebede B. Zewudie A. Mamo Y. (2020). Medication error and its contributing factors among pediatric patients diagnosed with infectious diseases admitted to Jimma University Medical Center, Southwest Ethiopia: prospective observational study. Integr. Pharm. Res. Pract. 9, 147–153. 10.2147/IPRP.S264941 32983947 PMC7501953

[B19] Feyissa Mechessa D. Dessalegn D. Melaku T. (2020). Drug-related problem and its predictors among pediatric patients with infectious diseases admitted to Jimma University Medical Center, Southwest Ethiopia: prospective observational study. SAGE open Med. 8, 2050312120970734. 10.1177/2050312120970734 33240498 PMC7675898

[B20] Gelchu T. Abdela J. (2019). Drug therapy problems among patients with cardiovascular disease admitted to the medical ward and had a follow-up at the ambulatory clinic of Hiwot Fana Specialized University Hospital: the case of a tertiary hospital in eastern Ethiopia. SAGE open Med. 7, 2050312119860401. 10.1177/2050312119860401 31367379 PMC6643177

[B21] Hepler C. D. Strand L. M. (1990). Opportunities and responsibilities in pharmaceutical care. Am. J. Hosp. Pharm. 47, 533–543. 10.1093/ajhp/47.3.533 2316538

[B22] Hoonhout L. H. De Bruijne M. C. Wagner C. Zegers M. Waaijman R. Spreeuwenberg P. (2009). Direct medical costs of adverse events in Dutch hospitals. BMC health Serv. Res. 9, 27–10. 10.1186/1472-6963-9-27 19203365 PMC2645386

[B23] Kaushal R. Bates D. W. Landrigan C. Mckenna K. J. Clapp M. D. Federico F. (2001). Medication errors and adverse drug events in pediatric inpatients. Jama 285, 2114–2120. 10.1001/jama.285.16.2114 11311101

[B24] Kearns G. L. Abdel-Rahman S. M. Alander S. W. Blowey D. L. Leeder J. S. Kauffman R. E. (2003). Developmental Pharmacology — drug disposition, action, and therapy in infants and children. N. Engl. J. Med. 349, 1157–1167. 10.1056/NEJMra035092 13679531

[B25] Maheshwari M. Sanwatsarkar S. Katakwar M. (2019). Pharmacology related to paediatric anaesthesia. Indian J. Anaesth. 63, 698–706. 10.4103/ija.IJA_487_19 31571682 PMC6761782

[B26] Meknonnen G. B. Biarra M. K. Tekle M. T. Bhagavathula A. S. (2017). Assessment of drug related problems and its associated factors among medical ward patients in university of gondar teaching hospital, northwest Ethiopia: a prospective cross-sectional study. J. Basic Clin. Pharma 8, 16–21.

[B27] Nascimento A. R. F. D. Leopoldino R. W. D. Santos M. E. T. D. Costa T. X. D. Martins R. R. (2020). Drug-related problems in cardiac neonates under intensive care. Rev. Paul. Pediatr. 38, e2018134. 10.1590/1984-0462/2020/38/2018134 31939506 PMC6958545

[B28] Nivya K. Kiran V. S. S. Ragoo N. Jayaprakash B. Sekhar M. S. (2015). Systemic review on drug related hospital admissions–A pubmed based search. Saudi Pharm. J. 23, 1–8. 10.1016/j.jsps.2013.05.006 25685036 PMC4310971

[B29] O'hara K. Wright I. M. Schneider J. J. Jones A. L. Martin J. H. (2015). Pharmacokinetics in neonatal prescribing: evidence base, paradigms and the future. Br. J. Clin. Pharmacol. 80, 1281–1288. 10.1111/bcp.12741 26256466 PMC4693494

[B30] Pantuzza L. L. Ceccato M. D. G. B. Silveira M. R. Junqueira L. M. R. Reis A. M. M. (2017). Association between medication regimen complexity and pharmacotherapy adherence: a systematic review. Eur. J. Clin. Pharmacol. 73, 1475–1489. 10.1007/s00228-017-2315-2 28779460

[B31] Pharmaceutical Care Network Europe Foundation (2010). Classification for Drug related problems. Available at: https://www.pcne.org/upload/files/11_PCNE_classification_V6-2.pdf.

[B32] Pharmaceuticals Fund and Supply Agency (2015). Standard operating procedures manual for the provision of clinical pharmacy services in Ethiopia. Addis Ababa, Ethiopia. Available at: https://siapsprogram.org/publication/standard-operating-procedures-manual-for-the-provision-of-clinical-pharmacy-services-in-ethiopia/.

[B33] Rashed A. N. Wilton L. Lo C. C. Kwong B. Y. Leung S. Wong I. C. (2014). Epidemiology and potential risk factors of drug‐related problems in H ong K ong paediatric wards. Br. J. Clin. Pharmacol. 77, 873–879. 10.1111/bcp.12270 24868576 PMC4004407

[B34] Schmidt S. J. Wurmbach V. S. Lampert A. Bernard S. Wilm S. Mortsiefer A. (2020). Individual factors increasing complexity of drug treatment—a narrative review. Eur. J. Clin. Pharmacol. 76, 745–754. 10.1007/s00228-019-02818-7 32239242 PMC7239823

[B35] Viktil K. K. Blix H. S. (2008). The impact of clinical pharmacists on drug-related problems and clinical outcomes. Basic & Clin. Pharmacol. Toxicol. 102, 275–280. 10.1111/j.1742-7843.2007.00206.x 18248511

[B36] Wong I. C. K. Wong L. Y. Cranswick N. E. (2009). Minimising medication errors in children. Archives Dis. Child. 94, 161–164. 10.1136/adc.2007.116442 18829622

[B37] World Health Organization (2002). WHO Policy Perspectives on Medicines - promoting rational use of medicines: core components. Geneva: World Health Organization. Available at: https://iris.who.int/bitstream/handle/10665/67438/WHO_EDM_2002.3.pdf.

[B38] World Health Organization (2013). Pocket book of hospital care for children. Available at: https://www.who.int/publications/i/item/978-92-4-154837-3. 24006557

[B39] World Health Organization (2019). Medication safety in polypharmacy: technical report. Geneva: World Health Organization. Available at: https://iris.who.int/bitstream/handle/10665/325454/WHO-UHC-SDS-2019.11-eng.pdf?sequence=1 .

